# Statistical process control and verifying positional accuracy of a cobra motion couch using step‐wedge quality assurance tool

**DOI:** 10.1002/acm2.12136

**Published:** 2017-07-21

**Authors:** Diana Binny, Craig M. Lancaster, Jamie V. Trapp, Scott B. Crowe

**Affiliations:** ^1^ Department of Radiation Oncology Cancer Care Services Royal Brisbane and Women's Hospital Queensland Australia; ^2^ Science and Engineering Faculty Queensland University of Technology Queensland Australia; ^3^ Present address: Diana Binny Radiation Oncology Centres Bayside Business Park 16‐24 Weippin Street Cleveland Qld 4163 Australia

**Keywords:** process analysis, quality assurance, SPC, tomotherapy, TQA

## Abstract

This study utilizes process control techniques to identify action limits for TomoTherapy couch positioning quality assurance tests. A test was introduced to monitor accuracy of the applied couch offset detection in the TomoTherapy Hi‐Art treatment system using the TQA “Step‐Wedge Helical” module and MVCT detector. Individual X‐charts, process capability (cp), probability (P), and acceptability (cpk) indices were used to monitor a 4‐year couch IEC offset data to detect systematic and random errors in the couch positional accuracy for different action levels. Process capability tests were also performed on the retrospective data to define tolerances based on user‐specified levels. A second study was carried out whereby physical couch offsets were applied using the TQA module and the MVCT detector was used to detect the observed variations.

Random and systematic variations were observed for the SPC‐based upper and lower control limits, and investigations were carried out to maintain the ongoing stability of the process for a 4‐year and a three‐monthly period. Local trend analysis showed mean variations up to ±0.5 mm in the three‐monthly analysis period for all IEC offset measurements. Variations were also observed in the detected versus applied offsets using the MVCT detector in the second study largely in the vertical direction, and actions were taken to remediate this error. Based on the results, it was recommended that imaging shifts in each coordinate direction be only applied after assessing the machine for applied versus detected test results using the step helical module. User‐specified tolerance levels of at least ±2 mm were recommended for a test frequency of once every 3 months to improve couch positional accuracy. SPC enables detection of systematic variations prior to reaching machine tolerance levels. Couch encoding system recalibrations reduced variations to user‐specified levels and a monitoring period of 3 months using SPC facilitated in detecting systematic and random variations. SPC analysis for couch positional accuracy enabled greater control in the identification of errors, thereby increasing confidence levels in daily treatment setups.

## 1 INTRODUCTION

Every high‐precision radiotherapy system requires pretreatment verification procedure based on existing quality control protocols and a method to enhance accuracy in patient positioning.^1^ Quality control (QC) protocols involve assessing if the machine performance is within specified tolerance levels and taking planned, systematic actions to ensure the same.^2^, ^3^, ^4^ QC of the treatment couch is important as it is a major component in patient localization during treatment, and various tests are recommended to ensure its optimal functioning at all times.^1^, ^4^, ^5^ TomoTherapy Hi‐Art (Accuray, Sunnyvale, CA, USA) units are equipped with a linear accelerator mounted on a CT gantry with an onboard detector for daily target verifications and software and hardware packages to regularly monitor its functional status.^1^, ^6^, ^7^ Any offsets from the planned position can be identified during pretreatment verifications and applied to correct for setup errors.

The couch alignment QC process in TomoTherapy requires comprehensive testing due to its additional capability of longitudinal translation during imaging/treatment compared with a conventional linear accelerator. This enables a higher degree of modulation for a specified target length. Dose to the patient is the integration of the longitudinal beam profile shaped with couch motion (ignoring leaf modulation), and hence any errors in couch motion would change delivered dose.^4^, ^8^, ^9^ TomoTherapy Quality Assurance (TQA) modules involve series of tests to ensure couch offsets, uniformity and speed, pitch, roll, yaw, sag, and beam profiles are within specified limitations.^4^, ^10^ Couch testing in some TQA modules involve the use of an aluminum step‐wedge placed on the couch and measuring variations in the beam attenuation from a reference to detect couch offsets for static and translational irradiations using the onboard megavoltage CT (MVCT) detector.

In order to ensure that the results of QC tests are within specified criteria, quality assurance (QA) tools like fault‐tree analysis, statistical process control (SPC) and other computational assessments have been applied in health care.^3^, ^11^, ^12^, ^13^, ^14^, ^15^, ^16^, ^17^, ^18^, ^19^ A few studies^1^, ^8^, ^9^, ^20^, ^21^ have been performed to test the mechanical and dosimetric capability of the high‐performance (HP) couch and other TomoTherapy machine parameters; however, there has been no studies that applied control charts to couch offset constancy measurements or verified positional accuracy of the HP couch at the isocenter.

In this study, we used SPC^11^ to 4 years of TQA step‐wedge measurement data to assess couch offset process limitations for different user‐specified action levels. SPC employs a process to convert data to information by using statistical techniques,^16^ in this case to identify random and systematic errors in the current process of couch testing using the aluminum step wedge in a helical beam. The control chart obtained from this technique shows how the process varies over time.^3^, ^12^, ^16^ A bold center line (CL) in this control chart corresponds to the average of the process which is also the reference for data point dispersion. The upper control limit (UCL) and lower control limit (LCL) indicate the range of the process, whereas points that are outside these limits indicate the process to be out of control. Investigations were carried out to assess these out of limit data points in this study.

The sensitivity of the HP couch was also tested by applying manual offsets from a verified reference position along the tomotherapy coordinate system.^4^

## 2 METHODS

### 2.A Machine characteristics

Measurement variations obtained from couch offset QC tests for two tomotherapy units (T1 and T2) were assessed in this study. An SPC‐based control chart was obtained from these data to assess limitations in couch movement using the TomoTherapy coordinate system. In this fixed machine coordinate system (also referred to as the IEC X, Y, and Z coordinate system in this study), for a patient lying head‐first supine on the couch, +x points toward patient left, +y points toward patient head, and +z points toward the patient anterior side.^4^, ^22^ Vertical movement on the HP couch is accomplished using an actuator (i.e., z‐axis actuator) that emulates a “cobra motion” where the couch moves toward the gantry when moving up and vice versa. The HP couch is fitted with encoders for controlling lateral, longitudinal, and vertical movement by sensing physical and electronic positional information and relaying it to the couch control assembly (CCA).

The TQA module “Step‐Wedge Helical” was used to obtain couch offset measurements for a 4‐year period. This TQA module (as described in Table 1) uses an aluminum step‐wedge positioned on the couch at the machine's virtual isocenter to obtain a series of measurements to assess the machine's functional status for a modulated helical beam and translating couch.^8^, ^22^, ^23^ Machine parameters such as energy, output, beam flatness and symmetry, couch IEC X, Y, and Z offsets, and gantry period are also measured during this test. The action level for couch offset analysis was set at ±2 mm. Action levels are also referred to as user‐specified upper and lower levels (USL/LSL) in this study.

**Table 1:** Description of set parameters of the step‐wedge helical module used in this study

Parameters	Step‐wedge helical
Jaw setting (cm)	1
MLC	Open
Couch speed (mm/s)	1
Number of gantry rotations	10
Beam on time (s)	200
Data compression factor	10
Purpose in this study	Testing IECX, Y and Z offsets

### 2.B IECX, Y and Z offset

The IECX offset is a calculated parameter that is determined from the baseline comparison of the center of the attenuation profile across the step‐wedge at 0 and 180‐degree gantry angles in the *x*‐axis. IECY offset is a baseline comparison of the location of the first step edge of a step‐wedge profile along *y*‐axis, and IECZ is calculated in a similar manner to IECX offset but using gantry angles 90° and 270°^10^ in the vertical direction or *z*‐axis. The offset study aimed at using the onboard detector to detect applied offsets in the patient/tomotherapy *x*,* y*, and *z* coordinate^24^ directions and assessing the corresponding changes on geometric and dosimetric parameters. The Step‐Wedge Helical module was run after applying each offset for this test. Gantry phase angle difference to IECX offset linearity was also assessed using the helical step‐wedge mode on both treatment units. Gantry phase angle difference measures how closely the current rotational data is in phase with reference data and is linearly proportional to IECX offset.^10^

In this study, step‐wedge position offsets were applied digitally using the position control panel (PCP) monitor along *x*,* y*, and *z* directions in increments of 0.5, 1, 2 and 5 mm in both positive and negative directions of the IEC coordinate system. Offsets were systematically applied starting from the positive direction followed by negative, each time measuring the detected offset and sending the step‐wedge back to its baseline position. This step was performed three times in each axis direction, and the results were reproducible to within ±0.2 mm. A piece of graph paper was placed at the same location as the step‐wedge to independently measure shifts to within ±0.2 mm accuracy. Actual versus detected offset, energy, output, and gantry phase angle variations were recorded. IEC offset variations are stated in terms of their absolute difference from reference positions in mm; energy and output variations are expressed in percentage and the gantry phase angle variations in terms of degrees (tolerance ±0.5°) from baseline. The user‐specified action level set for the IEC offset test was ±2 mm from baseline. This comparison is between applied and MVCT‐detected variations. This action level is not to be confused with the treatment couch mechanical (digital versus actual movement) tolerance of 1 mm.^5^ The uncertainty associated with step‐wedge measurements is ±1 mm^4^.

### 2.C Statistical process control

The stability of units T1 and T2 were assessed using SPC to determine if the discrepancies were of a systematic or random nature. Control charts were plotted for all IECX, Y, and Z offsets with CL, UCL, and LCL to compare the variations in the data with the range defined by control limits. When the data fall within the UCL and LCL, the process is said to be within control (with only random causes affecting the process) and out of control (due to systematic or nonrandom causes) when data points outside the range. For systematic causes, external influences are, therefore, required to bring the process back into control by identifying and removing these nonrandom causes from the process.^11^, ^12^ The number of observations for T1 and T2 were 1530 and 1388, respectively, over a 4‐year period. Conventionally, UCL and LCL are set at ±3 standard deviations from the center line implying that 99.7% of the data points would fall within the control points if the data were normally distributed.^14^ This study used individual X‐charts for all analyzed IEC offset measurements and plotted them as variation control charts as the dataset was a continuous set of observations^12^, ^14^; however, they are not normally distributed. The control limits were calculated from eqs. (1), (2), (3):^25^

(1) $$\text{UCL}=\bar{X}+3\frac{\bar{\mathrm{mR}}}{d_{2}\sqrt{n}}$$

(2) $$\text{CL}=\bar{X}$$

(3) $$\text{LCL}=\bar{X}-3\frac{\bar{\mathrm{mR}}}{d_{2}\sqrt{n}}$$

where R is the range of the group, $d_{2}$ is a constant and depends on the continuous set of n measurements. In this case, n is 1 and $d_{2}$ is 1.128.^10^ $\bar{mR}$ is the average of the moving range or the absolute values of the difference between two consecutive measurements (mR_i_ = $\left|x_{i}-x_{i-1}\right|$) and $\bar{X}$ is the mean of the dataset.

### 2.D Normal distribution test

For a normal or Gaussian distribution, goodness of fitness tests^26^, ^27^ are used to assess if the data collected show trends in their behavior or not. The Anderson–Darling^12^, ^27^, ^28^ (AD) statistic was used in this study to test the hypothesized distribution F(x) for normality according to the below equation:^27^, ^29^, ^30^

(4) $$A_{n}^{2}=-n-\sum_{i=1}^{n}\frac{2i-1}{n}\left[ln\left(FX_{i}\right))+ln\left(1-F\left(X_{n+1-i}\right)\right)\right]$$

where {X_1_ < … < X_n_} are the ordered sample data points and *n* is the number of data points in the data distribution. In the AD test, the decision to reject a null hypothesis (H_0_) is based on comparing the *P*‐value^31^ for the hypothesis (h) test with the specified significance level of 5% such that a *h* value of 0 would indicate that the distribution is normal and 1 otherwise. This test was performed on the 4‐year data and the three‐monthly data to test the observations for process capability and to detect local trends as described in the next section.

### 2.E Process capability analysis

Baseline comparisons for IEC offset measurements using the Step‐wedge Helical module were set at user‐specified action levels of ±2 mm and process indices for action levels of ±1 mm. Since there is no current protocol to adhere to for these limits, process indices $c_{p}$ and $c_{pk}$ were employed to quantify the process behavior. The process capability $c_{p}$ is used to compare the variation process of the data with respect to the upper and lower user‐specified limits relative to the dispersion of process data and is calculated from eq. (5).^14^

(5) $$c_{p}=\frac{\mathrm{USL}-\mathrm{LSL}}{6\sigma }$$

where USL and LSL are upper and lower user‐specified limits and *σ* is the standard deviation of the data distribution. A $c_{p}$ value of 1 would indicate that the process is within action limits, and a $c_{p}$ > 1 would mean that the process is well within specification limits. A $c_{p}$ value less than 1 indicates the process is outside a permissible range for a given action limit. However, in some cases, a high $c_{p}$ process can still be functioning poorly^11^, ^14^, ^16^; therefore, another index called process acceptability index $c_{pk}$ is also used to show how closely the process center is relative to the specified limit and is calculated from eq. (6) : 14

(6) $$c_{pk}=min\left(\frac{USL-X}{3\sigma },\frac{X-LSL}{3\sigma }\right)$$

As discussed earlier, $\bar{X}$ represents the center line, also known as the average process value. If the process is on target, the capability ratio will be equal to the acceptability ratio and a higher capability ratio relative to the acceptability ratio would mean that the process is not centered about the user‐specified action limits. Mean variations in the three‐monthly analysis were noted for all IEC X, Y, and Z directions. The normality, capability, and acceptability values with their corresponding probability were calculated from the measurement data using the MATLAB program (The MathWorks, Natick, NA, USA).

## 3 RESULTS

### 3.A Statistical process control and process analysis

**Figure 1:**
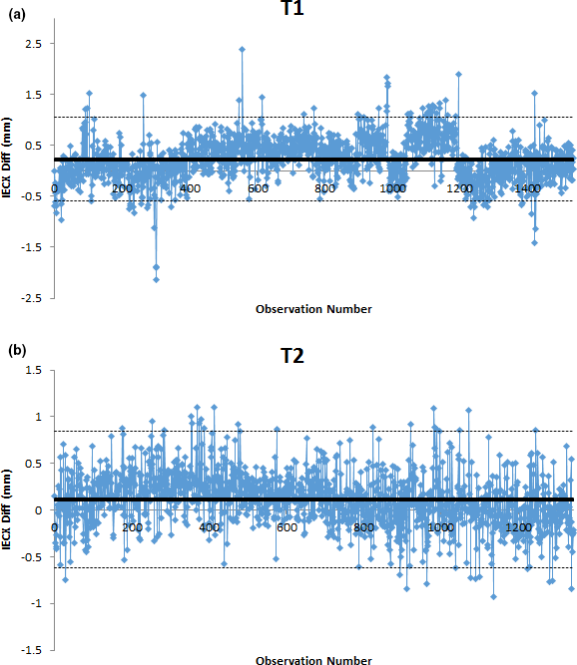
X‐control chart for a IECX offset measured for a 4‐year period for units T1 (a) and T2 (b).

The X‐charts for IEC X and Y offset evaluation for the two units T1 and T2 are shown in Figs. S1 and S2 (see Supplementary Material for IECY). Points outside the control limits were evaluated for both action limits of ±1 and 2 mm to examine the feasibility of the process being under control and if reducing limitations could be possible. Random and systematic variations were identified using SPC analysis for all offset measurements. Systematic variations in the data distribution were largely due to setup errors [Figs. 1(a) and 1(b)]. The IECX offset was measured along the transverse direction of the patient and found to be within process for the action limits specified.

Variations in the IECY offset were also observed to be within control limits and the user‐specified action limits for the analysis period considered for T1. T2, however, showed a period of systematic variations outside the SPC control limit in the observation period of 650–1100 (Fig. S1).

**Figure 2:**
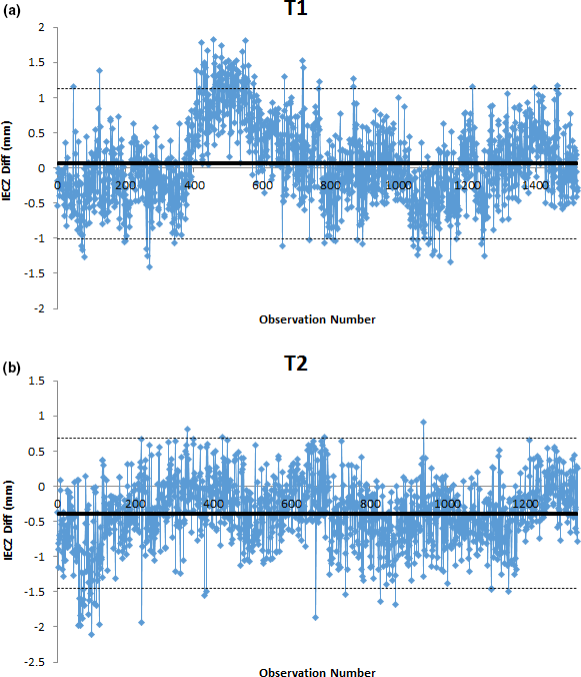
X‐control chart for a IECZ offset measured for a 4‐year period for units T1 (a) and T2 (b).

IECZ offset analysis showed the process to be out of control for T1 for the observation period 400–600. SPC analysis results for T2, however, were within control limits with a few errors due to setup indicated by points outside the UCL/LCL limits (Fig. 2). IECZ systematic variations were investigated for T1, and after consulting with the field specialist engineer and referring to machine logbooks, it was found that the Z‐axis encoder that provides angular feedback to the CCA enabling a closed loop control for its motion was faulty but still functioning within user‐specified action limitations. This fault was repaired and hence the variations disappeared from the process in the observation period post 600. T2 IECZ systematic variations were also investigated, and the variations were concluded to be due to setup errors (> 1 mm) from baseline positions after referring to machine logbooks.

**Figure 3:**
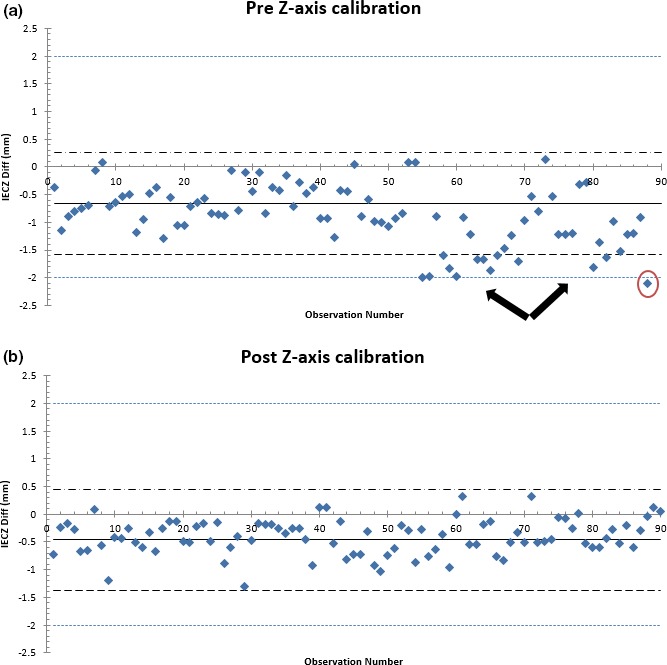
Retrospective (a) pre‐ and (b) post‐z‐axis encoder calibration measurements assessed using SPC for unit T2 for the first 180 observations. Black arrows indicate out of control points below the user‐specified limit of ±2 mm. Red circle indicates out of control point above ±2 mm action limit. Blue dashed lines represent the user‐specified limit of ±2 mm.

T2 IECZ axis offset measurements (normally distributed using AD test) were retrospectively assessed using SPC for the first 180 observations (Fig. 3). From machine logbooks, it was noted that the z‐axis encoder was calibrated when the system reported an out‐of‐tolerance measurement at the end of the first set of 90 observations. A subsequent SPC analysis showed that systematic variations were being observed prior to the system alert indicating that remediation to an out‐of‐control situation could have been made. The next set of measurements post the z‐axis encoder calibration indicated that the system was within the SPC calculated control limits. This further demonstrated the need for a statistical process control technique for machine QA.

**Figure 4:**
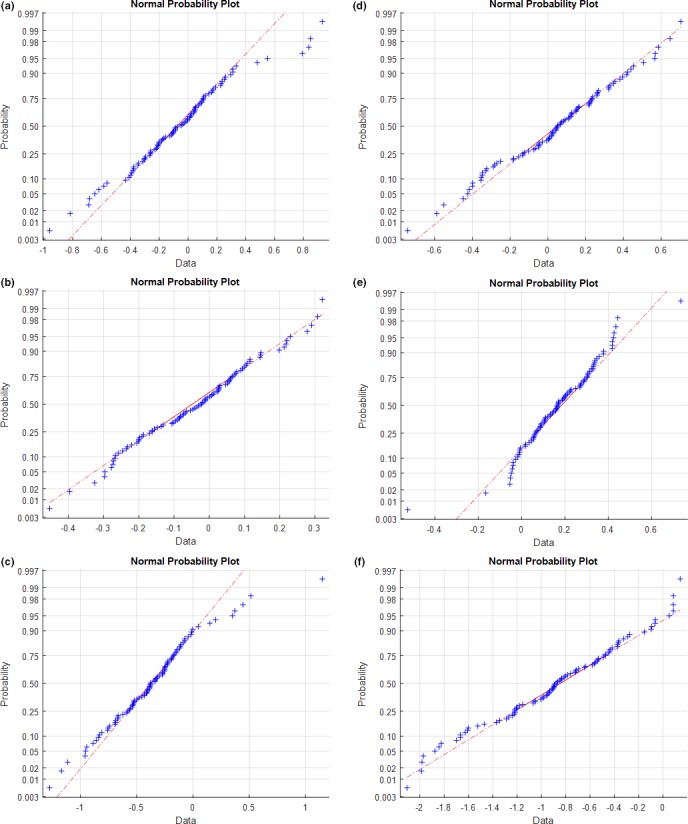
Normal distribution and probability analysis for units T1 and T2 using the Anderson–Darling test for a three‐monthly period.

Probability and process analysis was also performed for the two units for the 4‐year period (Fig. S2). Using the AD test, the three‐monthly observation data for both units showed a normal distribution for all IEC offset directions except X and Z for unit T1. See Fig. 4 and Table 2 for three‐monthly analysis results. The 4‐year analysis for both units did not show normality using the AD test.

**Table 2:** Process index values for process capability ($c_{p})$) and acceptability ($c_{\mathrm{pk}}$) for three‐monthly periods for units T1 and T2

SPC parameters	IEC offsets					
	T1			T2		
	X	Y	Z	X	Y	Z
UCL (mm)	0.650	0.3418	0.566	0.818	0.539	0.236
LCL (mm)	−0.775	−0.421	−1.298	−0.725	−0.192	−1.996
CL (mm)	−0.062	−0.039	−0.366	0.046	0.174	−0.880
*σ*	0.3436	0.165	0.377	0.295	0.171	0.542
AD^a^	Not normal	Normal	Not normal	Normal	Normal	Normal
No. of observations	90					

a Anderson–Darling test for normal data distribution.

**Figure 5:**
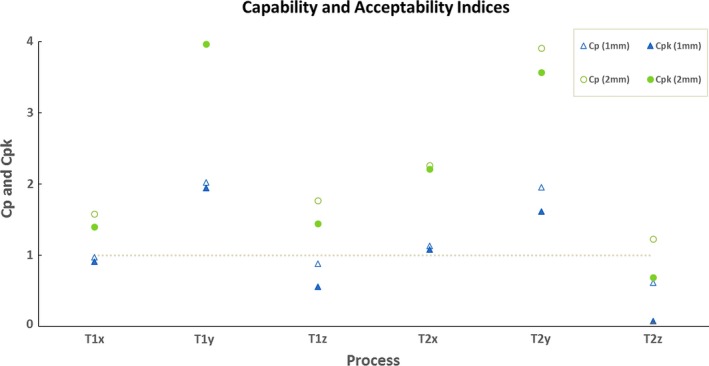
The capability ratio (c_p_) and acceptability ratio (c_pk_) for couch offset measurement analysis for action limits ±1 mm and ±2 mm for units T1 and T2 in the *x*,* y*, and *z* directions for a three‐monthly period. Values of c_p_ and c_pk_ above the dashed horizontal line were considered as acceptable.

Even though a precondition to use control charts is that the data must be normally distributed, this is not always the case.^32^ Control charts are designed to detect robustness of a process; however, during the initial phase of their use, the data may not be normally distributed. Figure 4 indicated that there were special causes (random or systematic) that may have led to non‐normal data distribution in the X and Z directions for unit T1. The results from process analysis (Supplementary Table S1 and Fig. S6 for 4‐year period and Table 2 and Fig. 5) showed that for USL/LSL = ±1 mm, process capability analysis for IECX and IECZ offset measurements are too conservative and are not feasible for the required outcome. Both units showed higher variations in the IECZ offset measurements for the period of 4 years and the test period of 3 months. Figure 5 for three‐monthly period in this article showed that the process was within limits for a user‐specified action limits of ±2 mm in most cases; however, from probability assessments, it was noted that the data distribution was not normal for IECZ and IECX offset measurements for the three‐monthly analysis for unit T1.

### 3.B IECX, Y and Z applied versus detected offset study

**Figure 6:**
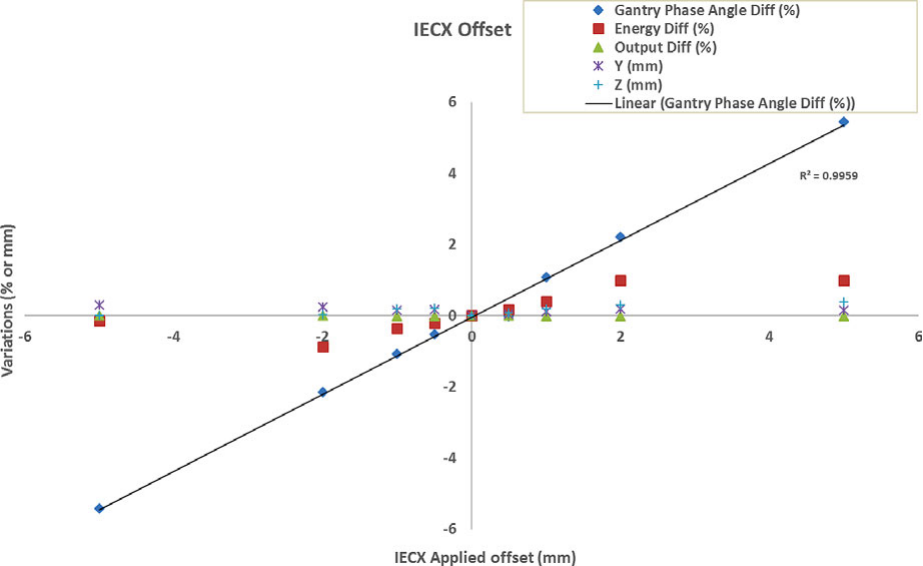
IECX applied offset versus variations of other detected parameters from baselines for unit T2.

**Figure 7:**
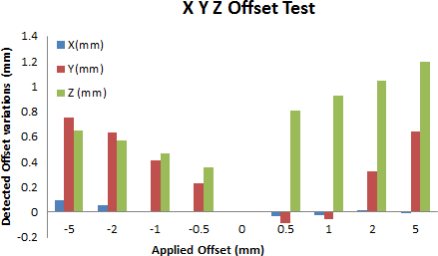
Applied versus detected offset values from the IECX, Y, and Z offset study.

Results from the manual offset study are shown in Figs. 6 and 7 and in Figs. S3–S5. Measured dosimetric and mechanical variations are also shown for each applied offset in this study.

As discussed in the TomoTherapy reference manual,^10^ a linear correlation (*R²* = 0.996) was seen between IECX offset and gantry phase angle. Any offset in the IECX direction did not affect the other coordinate (IECY or IECZ) to any >0.3 mm. Linear energy shifts from baselines were detected when lateral offsets were made in ≤±2 mm (*R* ^*2*^ = 0.996) and disappeared at ±5 mm. No explanation could be derived for this in this study. IECY and Z manual offsets did not report this behavior. These energy shifts are due to the applied offsets calculated by the onboard detector and not a change in the beam spectrum which was previously verified using the PDD method. Similar results were seen in the IECY offset test except the gantry phase angle variation was within ±0.31% for all applied offsets.

In the case of IECZ offset study, it was found that after each step‐wedge helical module was complete, the expected, corresponding shift was observed in the IECY direction and was also detected by the onboard MVCT detector. This is due to the cobra movement of the couch as discussed in the machine characteristic section. These shifts were only detectable for applied offsets ≥±2 mm (Fig. 7).

Figure 7 depicts the applied versus software detected offsets that were made during the IECX, Y, and Z offset study. It can be observed that the highest variation (+1.19 mm) was observed in the IECZ direction for a 5‐mm applied offset.

## 4 DISCUSSION

All baseline comparisons must adhere to user‐specified action limits set in the clinic after carrying out assessments using a quality control method for IEC offset values. Control limits that were calculated using SPC for process monitoring were lower than the action limits of ±2 mm in most cases (Fig. S6). We investigated individual cases where the process was observed to be out of control in this retrospective analysis from machine logbooks and by consulting with the engineer. Since no data were omitted past, the error was remediated; one can assume that the process could have a better signal‐to‐noise ratio if that step was carried out, i.e., a more stable process can be achieved for a desired control limit by identifying out of control points immediately as shown in Fig. 3. In most cases, it was observed that as the number of observations approached a three‐monthly period, variations in the X, Y, and Z offsets gradually shifted from their baseline value (mean variation up to ±0.5 mm). The standard deviation results for each analysis (4‐year period and three‐monthly test) have been shown in Table 2 and the supplementary material (Table S1). In both cases, the standard deviation is <0.57 mm. As mentioned earlier in the study, the out of control points in the local trend analysis may have been due to setup errors, laser, or couch encoder miscalibrations. Hence, as a conservative approach a three‐monthly process analysis of the IECX, Y and Z offset can be beneficial in identifying variations in machine behavior.

Geometric offsets of the treatment couch during the applied offset study were also calculated by the onboard detector and were within measurable accuracy of ±0.2 mm. IECZ coordinate variations were seen to affect *y* offset variations from reference up to a maximum deviation of 1.17 mm ±0.388 (SD) for the highest applied offset of 5 mm. The maximum deviations (>1 mm from applied offset) found in the IECZ study were reported to the engineer, and the *Z*‐axis encoding system was recalibrated. During this period, it was recommended to not apply any image shifts >2 mm in the vertical direction to reduce uncertainties in patient treatment until the error was resolved (Fig. 7).

Although mechanical and digital readout tests provide assurance in the couch position and movement, it is important to test the HP couch to assess the complete patient‐specific couch movements pre‐ and postdelivery/imaging as this can be particularly important in cases that involve treatment interruptions.

## 5 CONCLUSIONS

The TQA tool in TomoTherapy is an efficient system that provides a reliable overview of the machine dosimetric and geometric status. Long‐term data analysis and identifying control limits can be useful in separating random errors from systematic ones. Long‐term analysis of the IEC offset study showed that for user‐specified limits of ±2 mm, the process was within control and lowering this action threshold decreased process stability. IECZ axis offset analysis on both machines indicated a borderline acceptance according to the SPC capability analysis which led to a thorough investigation of the couch Z‐axis encoding system. Hence, we recommend carrying out SPC assessments and applied offset testing at least once every 3 months to regulate process capability, basing tolerances of ±2 mm to improve couch positioning accuracy and to detect any systematic errors in the process. The applied versus detected offset test can be used as an analysis method to detect variations in feedback mechanisms of the MVCT detector and other beam parameters. This will assist in identifying positional inaccuracies and provide greater confidence in patient setup.

## CONFLICT OF INTEREST

The authors declare no conflicts of interest.

## Supporting information


**Fig. S1:** X‐control chart for a IECY offset measured for a 4‐year period for units T1 (a) and T2 (b).
**Fig. S2:** Normal distribution and probability analysis for units T1 and T2 using the Anderson–Darling test for a 4‐year period.
**Fig. S3:** IECY applied offset versus variations of other detected parameters from baselines for unit T2.
**Fig. S4:** IECZ applied offset versus variations of other detected parameters from baselines for unit T2.
**Fig. S5:** Applies versus detected offsets in the IEC X, Y, and Z directions for unit T2.
**Fig. S6:** The capability ratio (*c*_p_) and acceptability ratio (*c*_pk_) for couch offset measurement analysis for action limits ±1 mm and ±2 mm for units T1 and T2 in the *x*,* y,* and *z* directions for a 4‐year period. Values of c_p_ and c_pk_ above the dashed horizontal line were considered as acceptable.
**Table S1:** Process index values for process capability (*c*_p_) and acceptability (*c*_pk_) for a 4‐year period for units T1 and T2.
