# Supplementary material for: Statistical process control and verifying positional accuracy of a cobra motion couch using step‐wedge quality assurance tool
Source: J Appl Clin Med Phys. 2017 Jul 21;18(5):70–9. doi: 10.1002/acm2.12136 (PMC5874965; doi:10.1002/acm2.12136)
Supplement: Supplementary file 1 — Fig. S1: X‐control chart for a IECY offset measured for a 4‐year period for units T1 (a) and T2 (b). Fig. S2: Normal distribution and probability analysis for units T1 and T2 using the Anderson–Darling test for a 4‐year period. Fig. S3: IECY applied offset versus variations of other detected parameters from baselines for unit T2. Fig. S4: IECZ applied offset versus variations of other detected parameters from baselines for unit T2. Fig. S5: Applies versus detected offsets in the IEC X, Y, and Z directions for unit T2. Fig. S6: The capability ratio (c p) and acceptability ratio (c pk) for couch offset measurement analysis for action limits ±1 mm and ±2 mm for units T1 and T2 in the x, y, and z directions for a 4‐year period. Values of cp and cpk above the dashed horizontal line were considered as acceptable. Table S1: Process index values for process capability (c p) and acceptability (c pk) for a 4‐year period for units T1 and T2. [file ACM2-18-70-s001.docx]

# **Supplementary material**


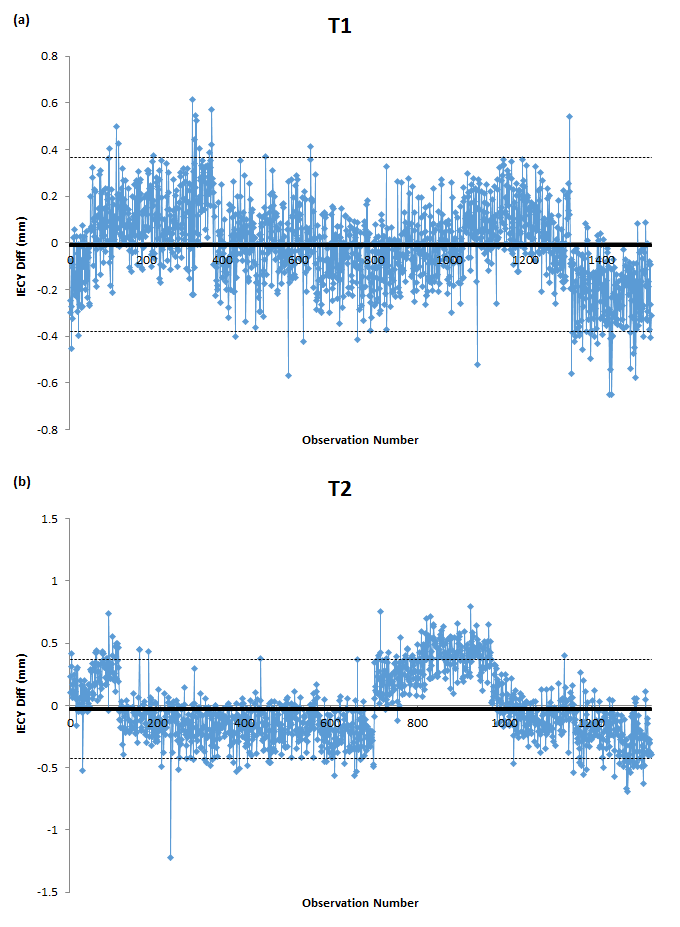


Figure 1. X-control chart for a IECY offset measured for a four-year period for units T1 (a) and T2 (b).


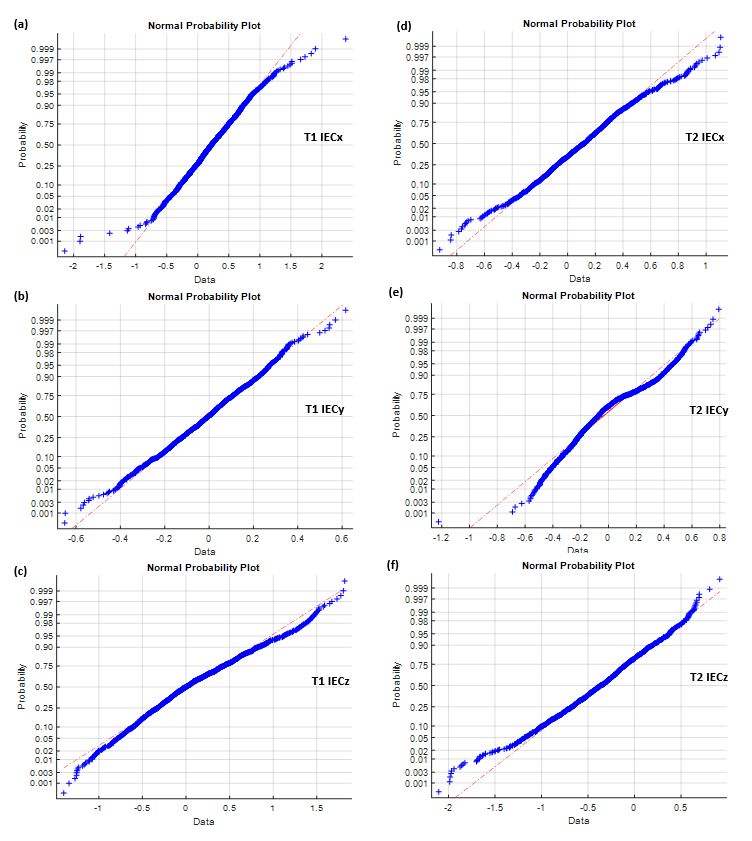


Figure 2. Normal distribution and Probability analysis for units T1 and T2 using the Anderson-Darling test for a four-year period.


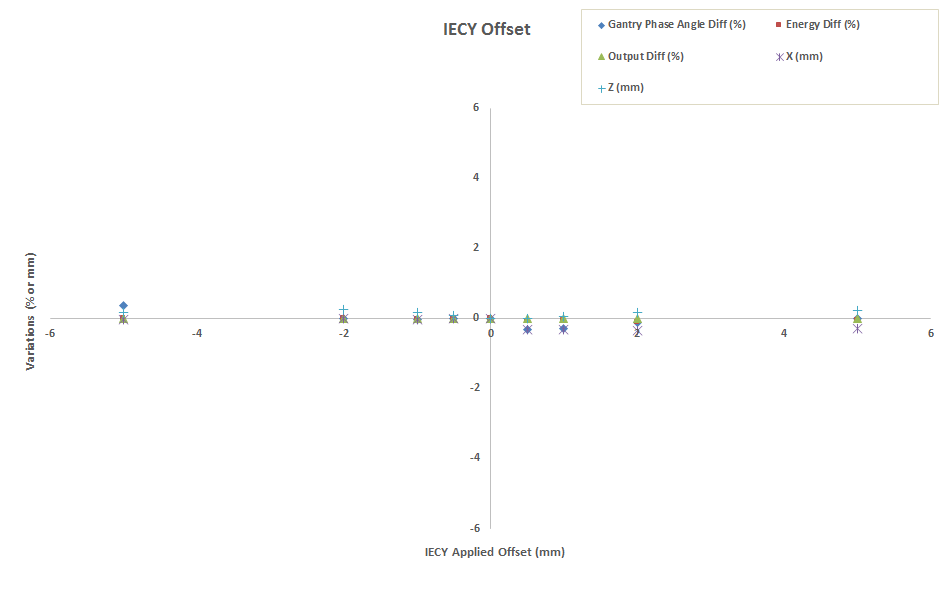


Figure 3. IECY applied offset versus variations of other detected parameters from baselines for unit T2.


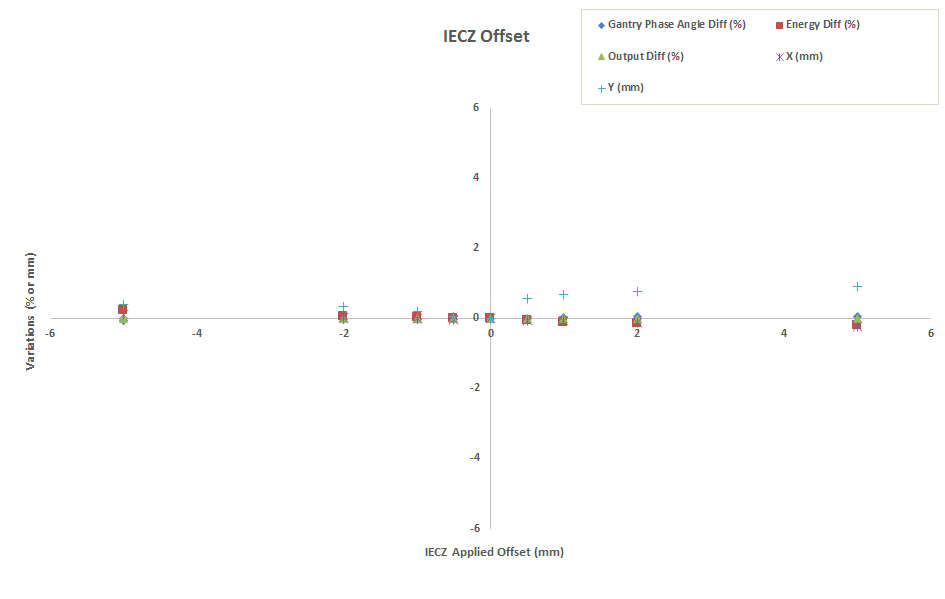


Figure 4. IECZ applied offset versus variations of other detected parameters from baselines for unit T2.


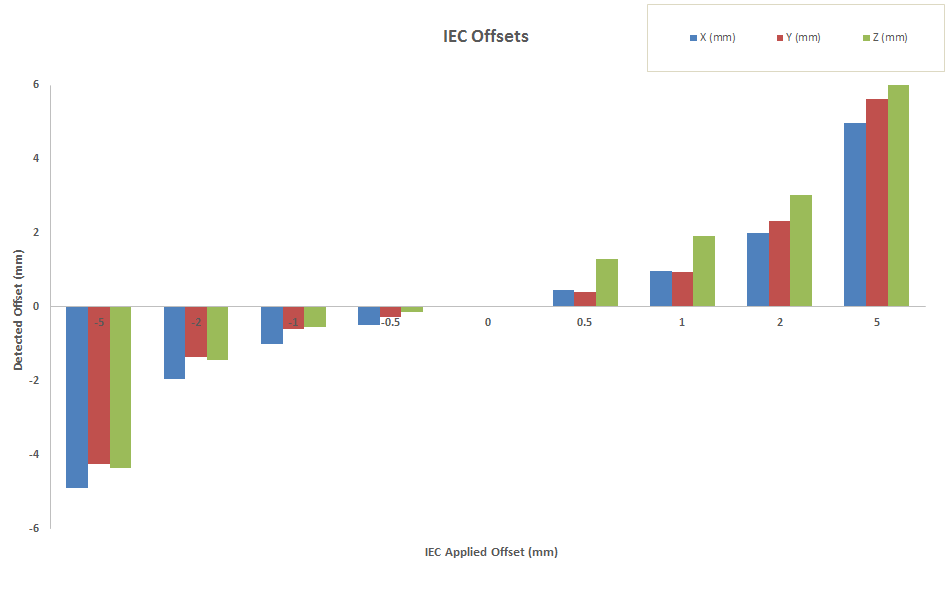


Figure 5. Applies versus detected offsets in the IEC X, Y and Z directions for unit T2.


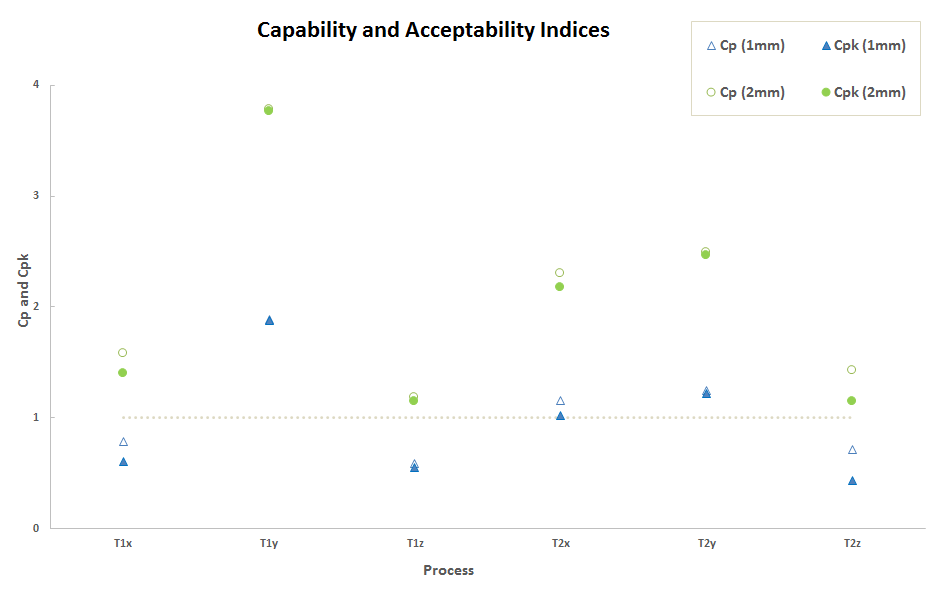


Figure 6. The capability ratio (c_p_) and acceptability ratio (c_pk_) for couch offset measurement analysis for action limits ±1 mm and ±2 mm for units T1 and T2 in the x, y and z directions for a four-year period. Values of c_p_ and c_pk_ above the dashed horizontal line were considered as acceptable.

Table 1. Process index values for process capability ($c_{p})$and acceptability ($c_{pk}$) for a four-year period for units T1 and T2.

|  |  |  |  |  |  |  |  |
| --- | --- | --- | --- | --- | --- | --- | --- |
| **SPC Parameters** | **IEC Offsets** | | | | | | |
|  | **T1** | | |  | **T2** | | |
|  | X | Y | Z |  | X | Y | Z |
| UCL (mm) | 1.050 | 0.366 | 1.132 |  | 0.843 | 0.372 | 0.683 |
| LCL (mm) | -0.593 | -0.382 | -1.010 |  | -0.618 | -0.424 | -1.456 |
| CL (mm) | 0.229 | -0.008 | 0.061 |  | 0.112 | -0.026 | -0.386 |
| σ | 0.4220 | 0.176 | 0.565 |  | 0.289 | 0.266 | 0.467 |
|  | **USL/LSL = ± 2 mm** | | | | | | |
| Cp | 1.579 | 3.781 | 1.179 |  | 2.303 | 2.496 | 1.425 |
| Cpk | 1.399 | 3.766 | 1.143 |  | 2.173 | 2.463 | 1.149 |
| P | 1.000 | 1.000 | 1.000 |  | 1.000 | 1.000 | 1.000 |
|  | **USL/LSL = ± 1 mm** | | | | | | |
| Cp | 0.790 | 1.890 | 0.589 |  | 1.151 | 1.248 | 0.712 |
| Cpk | 0.609 | 1.875 | 0.554 |  | 1.022 | 1.215 | 0.437 |
| P | 0.964 | 1.000 | 0.921 |  | 0.999 | 1.000 | 0.904 |
| No. of observations | 1530 | | |  | 1388 | | |
